# Studies on Growth Characteristics and Cross-Neutralization of Wild-Type and Delta SARS-CoV-2 From Hisar (India)

**DOI:** 10.3389/fcimb.2021.771524

**Published:** 2021-11-23

**Authors:** Nitin Khandelwal, Yogesh Chander, Ram Kumar, Himanshu Nagori, Assim Verma, Priyasi Mittal, Riyesh T, Sameer Kamboj, Sukhbir Singh Verma, Subhash Khatreja, Yash Pal, Baldev R. Gulati, Bhupendra N. Tripathi, Sanjay Barua, Naveen Kumar

**Affiliations:** ^1^ Virology Laboratory, National Centre for Veterinary Type Cultures, ICAR-National Research Centre on Equines, Hisar, India; ^2^ Civil Hospital, Hisar, India

**Keywords:** SARS-CoV-2, wild type, delta, antibody, cross-neutralization

## Abstract

Severe acute respiratory syndrome coronavirus 2 (SARS-CoV-2) has rapidly evolved to generate several antigenic variants. These variants have raised concerns whether pre-existing immunity to vaccination or prior infection would be able to protect against the newly emerging SARS-CoV-2 variants or not. We isolated SARS-CoV-2 from the coronavirus disease 2019 (COVID-19)-confirmed patients in the beginning of the first (April/May 2020) and second (April/May 2021) waves of COVID-19 in India (Hisar, Haryana). Upon complete nucleotide sequencing, the viruses were found to be genetically related with wild-type (WT) and Delta variants of SARS-CoV-2, respectively. The Delta variant of SARS-CoV-2 produced a rapid cytopathic effect (24–36 h as compared to 48–72 h in WT) and had bigger plaque size but a shorter life cycle (~6 h as compared to the ~8 h in WT). Furthermore, the Delta variant achieved peak viral titers within 24 h as compared to the 48 h in WT. These evidence suggested that the Delta variant replicates significantly faster than the WT SARS-CoV-2. The virus neutralization experiments indicated that antibodies elicited by vaccination are more efficacious in neutralizing the WT virus but significantly less potent against the Delta variant. Our findings have implications in devising suitable vaccination, diagnostic and therapeutic strategies, besides providing insights into understanding virus replication and transmission.

## Introduction

Severe acute respiratory syndrome coronavirus 2 (SARS-CoV-2), which emerged in 2019 in Wuhan, China, is responsible for the current pandemic of coronavirus disease 2019 (COVID-19) (Cao et al., 2020) and has resulted in more than 241.8 million infections and 4,919,755 deaths as of October 22, 2021 (WHO, 2021). Currently, there is no specific antiviral drug to treat COVID-19. However, vaccine is available, and currently, the world’s 7.9 billion population is being vaccinated to generate herd immunity. The original SARS-CoV-2, first detected in Wuhan, has undergone extensive mutations and has resulted in the generation of several antigenic variants. The major antigenic variants include Alpha, Beta, Gamma, and Delta and are believed to have originated in UK (December 2020), South Africa (September 2020), Brazil (November 2020), and India (May 2021), respectively (Duong, 2021). Currently, SARS-CoV-2 Delta variant (B.1.617.2) is predominantly circulating in India and several other parts of the world (Noh et al., 2021; Sanyaolu et al., 2021; Winger and Caspari, 2021). It has been classified as a variant of concern and is believed to be 60% more transmissible than the Alpha variant (B.1.1.7) (Duong, 2021). New variants of interest are Eta, Iota, Kappa, Lambda, and Mu, and many more are likely to emerge (Areo et al., 2021; Janik et al., 2021; Parums, 2021). The emergence and widespread prevalence of new SARS-CoV-2 variants can reduce the effectiveness of the current vaccines and pose a significant threat to combat the pandemic (Becker et al., 2021; Duong, 2021; Ferraz et al., 2021; Sharun et al., 2021). Besides vaccine efficacy, genetic/antigenic variations may also affect the capability of the diagnostic tests, which are based on WT SARS-CoV-2. Besides, there is a significant gap in our understanding about the life cycle of the wild-type (WT) and Delta strains of SARS-CoV-2. This study provides a comparative insight on the growth characteristics and cross-neutralization of WT and Delta strains of SARS-CoV-2.

## Materials and Methods

### Cells

Vero cells were available at the National Centre for Veterinary Type Cultures (NCVTC), Hisar, and grown in Dulbecco’s modified Eagle’s medium (DMEM) supplemented with antibiotics and 10% fetal calf serum.

### Nasopharyngeal Swabs and Serum Samples

Nasopharyngeal swabs were received from the Civil Hospital Hisar, Haryana (India). Serum samples from the vaccinated and/or COVID-19-positive individuals were also received from the Civil Hospital, Hisar. Depending on the exposure to SARS-CoV-2, serum samples were categorized into the following types, namely, infected during the first wave (W1), infected during the second wave (W2), uninfected but vaccinated (V), infected during the first wave and subsequently vaccinated (W1V), and infected during the second wave and later vaccinated (W2V). Paired samples were also collected from an individual who succumbed to COVID-19 during both the first and second waves (W1W2).

### Ethics Statement

Samples were collected from patients by the authorized District Medical Officer Hisar, India. A due consent was taken from the patients before collection of the serum samples.

### Virus Isolation

SARS-CoV-2 was propagated in Vero cells in the Biosafety Level 3 (BSL-3) laboratory of ICAR-National Research Centre on Equines (ICAR-RCE), Hisar. The work was approved by the Institute Biosafety Committee and Review Committee on Genetic Manipulation (RCGM), Department of Biotechnology, Government of India (No. BT/BS/17/436/2011-PID).

Virus isolation was attempted from the nasopharyngeal swabs received for COVID-19 testing at our facility at ICAR-NRCE Hisar. Nasopharyngeal swabs that were positive for SARS-CoV-2 genome with a cycle threshold (cT) value of <20.0 in quantitative real-time PCR (qRT-PCR) were considered for virus isolation in Vero cells. Samples that produced cytopathic effect (CPE) within three successive blind passages in Vero cells were authenticated and accessioned. Samples that did not produce CPE up to the third blind passage were discontinued. The first attempt of virus isolation was made in April 2020, which was the beginning of COVID-19 pandemic in Haryana (India). Similarly, virus isolation was also attempted from nasopharyngeal swabs (n = 11) that were collected following the onset of the second wave in India (April/May 2021).

### Identification

The viral RNA was extracted using QIAmp Viral RNA Mini Kit (Qiagen, Hilden, Germany). This was followed by amplification of SARS-CoV-2 RdRp and E gene using LabGun COVID-19 Assay (LabGenomics Co., Ltd., Suwon-si, Republic of Korea) using CFX96 PCR Detection Systems (BioRad, USA).

### Complete Genome Sequencing of the Viral Isolates

Viral RNA was extracted by TRIzol Reagent (Invitrogen, CA, USA). Complementary DNA (cDNA) was synthesized as per the protocol described by the manufacturer (Fermentas, Hanover, USA) and sent to Clevergene Biocorp Pvt Ltd. (Bengaluru, India) for complete genome sequencing. Briefly, first-strand cDNA reactions were converted to double-stranded DNA (dsDNA). The double-stranded cDNA fragments obtained were cleaned up by using 1.8× of AMPure XP beads (Catalog no. A63881, Beckman Coulter). The purified cDNA was run on the tape to check the fragment size of cDNA. The library concentration was determined in a Qubit.3 Fluorometer (Catalog no. Q33216, Life Technologies) using the Qubit dsDNA High Sensitivity Assay Kit (Catalog no. Q32854, Thermo Fisher Scientific). The library quality assessment was done using Agilent D5000 Screen Tape System (Catalog no. 5067-5588, Agilent) in a 4150 Tape Station System (Catalog no. G2992AA, Agilent).

The sequence data were generated using Illumina HiSeq. Data quality was checked using FastQC (Ewels et al., 2016) software. Raw sequence reads were processed to remove adapter sequences and low-quality bases using fastp (Chen et al., 2018). To make consensus sequences, quality trimmed were aligned to respective genomes transcriptome using bwa-mem. The mean depth was calculated using mosdepth (Pedersen and Quinlan, 2018) and sambamba (Tarasov et al., 2015). From the aligned bam files, variants were called with –ploidy 1 option, and consensus genome sequences were built using the reference genome using bcftools from the samtools package (Li et al., 2009). SnpEff v 4.1 was used to annotate the variants (Cingolani et al., 2012) with respect to their reference sequence.

The complete nucleotide sequences of WT and Delta strains reported in this study were compared with the original SARS-CoV-2, reported for the first time in late 2019 in Wuhan, China (GenBank Accession Number MN996528.1), and with a reference Delta strain (GenBank Accession Number OK091006.1), and the mutations were identified by using an online server (https://www.gisaid.org).

### Plaque Assay

SARS-CoV-2 plaque assay was performed as described previously (Kumar et al., 2018; Kumar et al., 2019). Briefly, the confluent monolayers of Vero cells were infected with 10-fold serial dilutions of SARS-CoV-2 for 1 h at 37°C, after which the infecting medium was replaced with an agar overlay containing equal volume of 2× L-15 medium and 1% agar. Upon development of plaques, the agar overlay was removed, and the plaques were stained by 1% crystal violet.

### Virus Neutralization Assay

The virus neutralization assay was carried out as per the previously described method with some modifications (Kumar et al., 2020; Kumar et al., 2021). Serum samples were initially heated at 56°C for 30 min to inactivate the complement. Vero cells were grown in 96-well tissue culture plates at ~90 confluency. Twofold serum dilutions (in 50 µl volume) were made in phosphate-buffered saline (PBS) and incubated with equal volume of SARS-CoV-2 (10^4^ PFU/ml) for 1 h. Thereafter, a virus–antibody mixture was used to infect Vero cells. The cells were observed daily for the appearance of the CPE. Final reading was taken at 48 h postinfection (hpi) (SARS-CoV-2 Delta) or at 72 hpi (SARS-CoV-2 WT) for the determination of antibody titers.

### One-Step Growth Curve

Confluent monolayers of Vero cells, in triplicates, were infected with SARS-CoV-2 at multiplicity of infection (MOI) of 5 and thereafter washed with PBS, and fresh MEM was added. Infectious progeny virus particles released in the cell culture supernatant at various time points were quantified by plaque assay.

### Kinetics of SARS-CoV-2 Genome Synthesis (qRT-PCR)

Confluent monolayers of Vero cells, in triplicates, were infected with SARS-CoV-2 at MOI of 5, followed by washing with PBS and addition of fresh MEM. Cells were scrapped at indicated time points and subjected to RNA extraction and quantitation of viral RNA by qRT-PCR as describe above. cT values were normalized with β-actin [primers describe elsewhere (Khandelwal et al., 2020)] housekeeping control gene, and relative fold change in RNA copy numbers was calculated by ΔΔCt method (Holmes et al., 2010).

## Results

### Complete Genome Sequencing (Genotyping)

During the first wave of COVID-19, 11 nasopharyngeal swabs from COVID-19-confirmed patients were subjected to complete genome sequencing. Their sequences are available in GenBank with Accession Numbers, namely, MW555317, MW555320, MW555325, MW555334, MW555576, MW555595, MW555597, MW555280, MW927136, MW555786, and MW555598. Upon comparison of the nucleotide/amino acid sequences, rather than any variants of concern (observed later during the pandemic), these viruses were found to be more closely related with the original SARS-CoV-2 reported during the early stage of pandemic in China. Hereinafter, we refer them as wild-type (WT) SARS-CoV-2 strain(s), although only one of them was used for the detailed investigation in this study. Six amino acid mutations (**Table 1**) were observed in the WT as compared to the Wuhan SARS-CoV-2.

**Table 1:** Mutational analyses of WT and Delta strains of SARS-CoV-2.

Sr. No.	Gene Name	WT*	Delta (Reference Strain)	Delta*
1.	NSP3	T1198K	A488S V932A P1228L P1469S	A488S H795Y P1228L P1469S
2.	NSP4		V167L T492I	V167L T492I
3.	NSP5			S123F V296I
4.	NSP6	L37F M83I	T77A	T77A
5.	NSP12	A97V	P323L G671S	P323L G671S
6.	NSP13		P77L	P77L
7.	NSP14		A394V	A394V
10.	Spike		T19R **G142D** **L452R** **T478K** **D614G** **P681R** **D950N**	T19R **G142D** **L452R** **T478K** **D614G** **P681R** **D950N** T95I P812R
11.	NS3		S26L	S26L
13.	NS7a		V82A	V82A T120I
14.	NS7b		T40I	T40I
15.	NS8	E106Q	F120L	
16.	M			I82T
17.	N	P13L	D63G R203M G215C D377Y	D63G R203M G215C D377Y S79I

Comparisons were made with a reference strain (GenBank Accession Number MN996528.1, reported in the beginning of the COVID-19 epidemic in Wuhan, China). GenBank Accession Number OK091006.1-a Delta reference strain was also included in the study. Bold letters represent signature mutations of the Delta variant.

*SARS-CoV-2 strains belong to this study.

Likewise, three virus isolates from the second wave (April/May 2021) were also subjected to complete genome sequencing (sequence will be provided on request). On BLAST search, these sequences were found to be closely related with the Delta variants of SARS-CoV-2 and possess all the Delta-specific signature mutations (**Table 1**). One of the Delta strains (employed for detailed investigations in this study) that was compared with the reference Delta strain showed six amino acid mutations (**Table 1**).

### Virus Isolation

The first COVID-19-positive case in Haryana was reported from the Gurugram district on March 17, 2021. By April 2020, it had spread to almost the entire state. Our laboratory started testing for COVID-19 from April 12, 2020. Some of the samples that were received in April/May 2020 and had a cT value of <20 were subjected to virus isolation. In April/May 2020, out of the 11 nasopharyngeal swabs subjected to virus isolation, only 4 produced CPE up to the third blind passage, 3 of which were further authenticated and deposited with accession numbers of VTCCAVA 294 (SARS-CoV-2/India/2020/tc/Hisar/4907), VTCCAVA295 (SARS-CoV-2/India/2020/tc/Hisar/2710), and VTCCAVA296 (SARS-CoV-2/India/2020/tc/Hisar/1469) at the National Repository of Animal Microbes (NCVTC, Hisar, Haryana; www.ncvtc.org.in). The virus with accession number VTCCAVA295 was used as a prototype of WT SARS-CoV-2 for various biological assays described in this study.

On the onset of the second wave of COVID-19 (April/May 2021) in India, we again attempted virus isolation from the samples received from the Hisar district of Haryana. Out of the 11 nasopharyngeal swabs, 1 swab sample produced CPE even on the first blind passage, whereas 2 produced CPE during the second blind passage. These three virus isolates were further authenticated and deposited at the repository described above with accession numbers, namely, VTCCAVA318 (SARS-CoV-2/India/2021/tc/Hisar/177124), VTCCAVA319 (SARS-CoV-2/India/2021/tc/Hisar/177405), and VTCCAVA320 (SARS-CoV-2/India/2021/tc/Hisar/177961). The virus with accession number VTCCAVA319 was later used as a prototype of Delta variant of SARS-CoV-2 for various biological assays described in this study.

**Figure 1:**
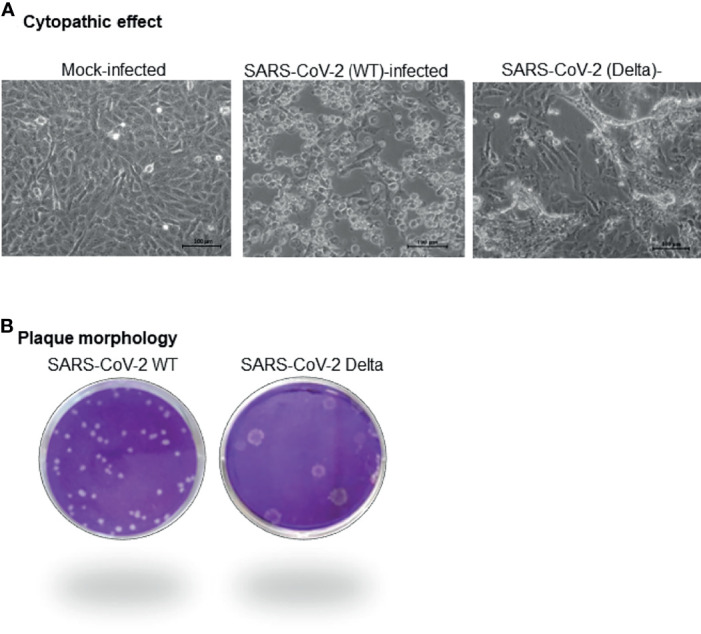
Virus isolation. Nasopharyngeal swabs positive for COVID-19 with cT value of <20.0 in qRT-PCR were considered for virus isolation in Vero cells. The samples were filtered in a 0.45-µm syringe filter, and 500 µl of the filtrate was used to infect Vero cells. Samples that produced CPE within three successive passages in Vero cells were authenticated and accessioned. Characteristics of the CPE produced by WT and Delta **(A)** variant of SARS-CoV-2 at passage level 5 is shown. Plaque morphology of WT and Delta variant of SARS-CoV-2 is also shown **(B)**.

At passage level 5 (Vero cells), whereas WT SARS-CoV-2 took 3–4 days in producing appreciable CPE in Vero cells, the Delta variant was able to produce significant cell death within 24–36 h, suggesting a higher replication rate of the Delta variant. Besides, the nature of the CPE was also strikingly different; whereas infection of WT virus resulted in cell rounding, detachment, degeneration, and occasionally small syncytia (**Figure 1A**), the Delta variant produced elongated and extremely large syncytia, besides inducing degeneration and detachment of the cells (**Figure 1A**). Most importantly, plaques produced by the Delta variant were much larger in size as compared to the WT SARS-CoV-2 (**Figure 1B**).

### SARS-CoV-2 Life Cycle (One-Step Growth Curve)

**Figure 2:**
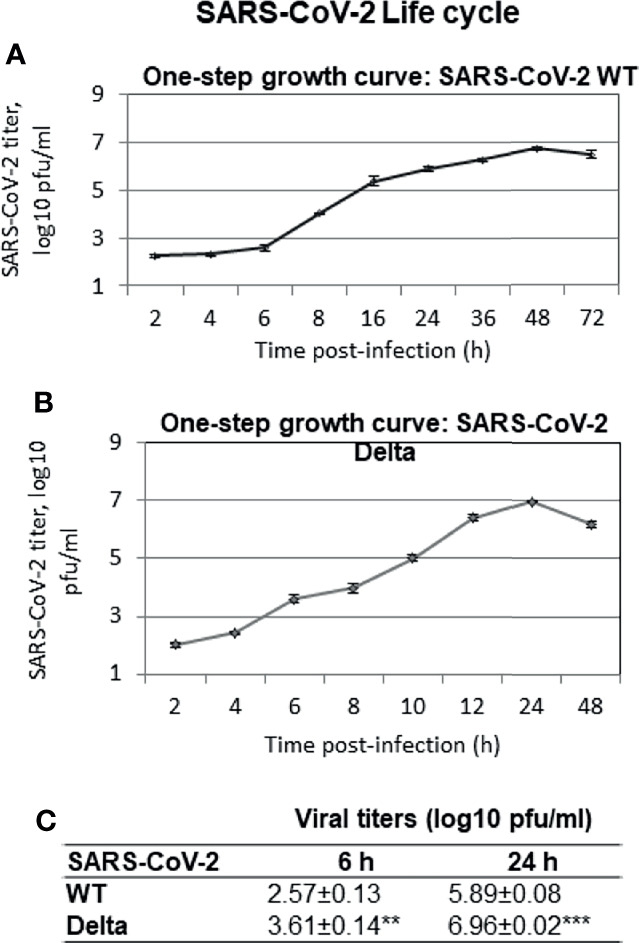
SARS-CoV-2 life cycle. Confluent monolayers of Vero cells, in triplicates, were infected with SARS-CoV-2 at MOI of 5 and thereafter washed with PBS, and fresh MEM was added. Infectious progeny virus particles released in the infected cell culture supernatant at indicated time points were quantified by plaque assay. One-step growth curve of WT **(A)** and Delta variant **(B)** of SARS-CoV-2 is shown. Statistical comparisons of viral titers (WT versus Delta SARS-CoV-2) were performed at 6 hpi (when progeny virus particles start appearing in the infected cell culture supernatant) and at 24 hpi (when Delta virus is about to complete its life cycle) by two*-*tailed Student’s t-test **(C)**. **p < 0.01, ***p < 0.001.

In order to determine the length of the viral life cycle, Vero cells were infected with high MOI (MOI = 5), and the virus released in the infected cell culture supernatant at different times postinfection was quantified. There was no significant difference in the vial titers in the infected cell culture supernatant that were collected at 2 h postinfection (hpi) and 4 hpi in both WT (**Figure 2A**) and Delta (**Figure 2B**) strains. However a sudden increase in viral titers was observed at 8 and 6 hpi, respectively in WT (**Figure 2A**) and Delta (**Figure 2B**) strains. This increase in viral titers was presumably due to the appearance of infectious progeny virus particles in the infected cell culture supernatant and hence indicated the completion of viral life cycle at these time points. The higher viral titers in Delta as compared to the WT SARS-CoV-2-infected cells at 6 hpi (**Figure 2C**) suggested that Delta variant has a significantly shorter life cycle than the WT SARS-CoV-2. Although the peak viral titers (~10^7^ pfu/ml) were almost similar in the supernatant collected from both WT (**Figure 2A**) and Delta (**Figure 2B**) strains, this was achieved significantly faster in Delta (~ 24 hpi) as compared to the WT strain (48 hpi) of SARS-CoV-2 (**Figure 2C**).

**Figure 3:**
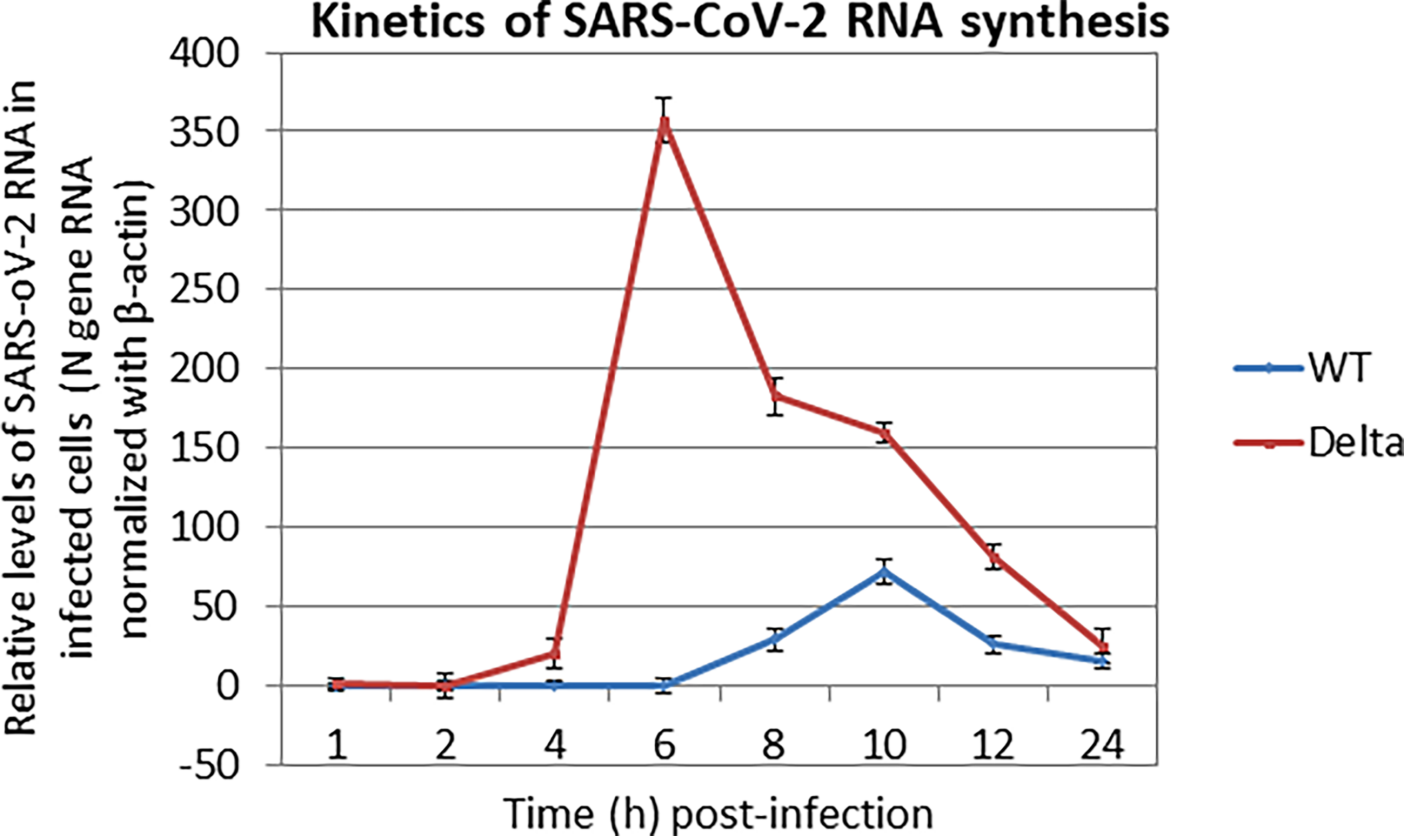
Kinetics of SARS-CoV-2 RNA synthesis. Confluent monolayers of Vero cells, in triplicates were infected with SARS-CoV-2 at MOI of 5, followed by washing with PBS and addition of fresh MEM. Cells were scrapped at indicated time points and subjected for the quantitation of SARS-CoV-2 RNA (N gene) by qRT-PCR. cT values were normalized with β-actin housekeeping control gene, and relative % fold change in viral RNA copy numbers at various time points (as compared to 1 hpi) was calculated by ΔΔCt method.

We also examined the kinetics of the SARS-CoV-2 RNA synthesis in cultured cells wherein Vero cells were infected with high MOI (MOI = 5), followed by quantifying the levels of SARS-CoV-2 RNA in the cell pellet at different times postinfection. A peak level of viral RNA was observed at ~9 h and ~6 h (**Figure 3**), respectively, in WT and Delta variant of SARS-CoV-2, which again suggested the faster replication (fitness) rate of the Delta variant.

### Cross-Neutralization Between WT and Delta Variants of SARS-CoV-2

We performed a virus neutralization assay in order to determine whether the sera from the COVID-19 vaccine recipient (Covishield/Covaxin used in India) and those who recovered naturally from the disease are able to neutralize the variants of concerns. There was a poor cross-reactivity between the sera derived from V/W1/W1V individuals with Delta variants; the antibody titers were in the range of 16–256 and 0–16 when neutralized with WT and Delta variant, respectively (**Table 2**). Likewise, sera derived from W2 individuals more effectively neutralized the Delta (titers range from 32 to 128) as compared to the WT SARS-CoV-2 (titers range from 0 to 32) (**Table 2**). The W2V individuals had higher antibody levels against both Delta and WT virus as expected. Exceptionally, two of the V-type sera (**Table 2**; SR134 and SR139) from diabetic patients were equally neutralized by both WT and Delta variants. Interestingly, one of the individuals (SR116A and SR116B, **Table 2**) developed clinical disease and tested positive for COVID-19 during both the first and second waves. Initially, 29 days following primary infection, the antibody titers in this individual were 32 and <8, respectively, against WT and Delta SARS-CoV-2, whereas at 18 days following secondary infection, the titers were 64 and 128, respectively.

**Table 2:** Cross-neutralization of SARS-CoV-2 WT and Delta variant.

	Serum ID	Infection/Vaccination status	Sample History	Vaccine type	Ab titer (WT)	Ab titer (Delta)	Significance (T test)
History of exposure to WT SARS-CoV-2	SR-6	W1	Day 15	NA	16	8	p < 0.041
	SR-8	W1	Day 18	NA	32	<8	
	SR-9	W1	Day 10	NA	16	8	
	SR-13	W1	Day 14	NA	32	0	
	SR-14	W1	Day11	NA	32	8	
	SR-17	W1	Day 12	NA	16	8	
	SR-18	W1	Day 14	NA	32	0	
	SR-19	W1	Day 15	NA	32	16	
	SR-21	W1	Day 10	NA	<8	<8	
	SR-116A*	W1	Day 29	NA	32	<8	
	SR-119	W1V	Day 98 (2nd dose)	Covishield	256	16	
	SR-125	V	Day 40 (2nd dose)	Covishield	32	16	
	SR-127	W1	Day 105	NA	128	32	
	SR-132	V	Day 42 (2nd dose)	Covishield	16	16	
	SR-133	V	Day 22 (1st dose)	Covishield	16	8	
	SR-134	V	Day 48 (2nd dose)	Covishield	128	128	
	SR-135	V	day 19 (1st dose)	Covishield	16	0	
	SR-136	V	Day 30 (2nd dose)	Covaxin	16	<8	
	SR-137	V	Day 109 (2nd dose)	Covishield	32	8	
	SR-138	V	Day 28 (2nd dose)	Covishield	32	16	
	SR-139	V	Day 10 (2nd dose)	Covaxin	128	128	
	SR-140	V	Day 57 (2nd dose)	Covishield	16	8	
	SR-141	V	Day 62 (2nd dose)	Covishield	128	64	
	SR-142	V	Day 58 (2nd dose)	Covishield	16	<8	
	SR-143	V	Day 58 (2nd dose)	Covishield	32	16	
	SR-144	V	Day 84 (2nd dose)	Covishield	32	16	
History of exposure to Delta SARS-CoV-2	SR-126	W2	Day 45	NA	32	128	p < 0.035
	SR-128	W2	Day 51	NA	32	128	
	SR-129	W2	Day 22	NA	16	32	
	SR-130	W2	Day 56	NA	16	64	
	SR-131	W2	Day 37	NA	<8	32	
Exposure to both WT and Delta variant	SR-116B*	W1W2	Day 18 (2nd infection)	NA	64	128	NA
	SR-124	W2V	Day 34 (1st dose)	Covishield	64	64	

Pairwise statistical comparison of antibody titers in serum samples (WT versus Delta variant) was performed by two-tailed Student’s t-test. For statistical analysis, antibody titer <8 was considered as 8.

W1, infection during the first wave; W2, infection during the second wave; V, uninfected but vaccinated; W1V, infected during the first wave and then vaccinated; W2V, infection during the second wave and later vaccinated; Ab, antibody; NA, not applicable.

*The individual infected during both the first and second waves (SR116A, sample collected at 29 days post-first wave infection; SR116B, sample collected at 18 days post-second wave infection).

## Discussion

Vaccinating the world’s 7.9 billion population against COVID-19 is a huge challenge (Asawapaithulsert et al., 2021; Eichhorst, 2021; Perkins, 2021). As on September 2, 2021, only 40.1% of the world population has received at least one dose of the vaccine, while 38.92 million are now vaccinated each day. Only 1.8% of people in low-income countries have received at least one dose (Organization, 2021). Most of the vaccines being used worldwide are based on the SARS-CoV-2 strain(s) isolated in patients in December 2019 or early 2020. Since the virus rapidly undergoes mutations, a lot of antigenic variants have been reported. Currently, the major variants of concerns are Alpha, Beta, Gamma, and Delta (Lessells, 2021; Noh et al., 2021; Sander et al., 2021). New variants of interest are Eta, Iota, Kappa, Lambda, and Mu (Areo et al., 2021; Janik et al., 2021; Parums, 2021). New variants are believed to have more transmissibility and produce more lethal disease (Gravagnuolo et al., 2021). Most of these variants harbor mutations in the spike protein, thereby raising concerns whether pre-existing antibodies due to vaccination or infection (recovered individuals) would be able to protect against the newly emerging variants or not (Sharun et al., 2021). Besides vaccine efficacy, genetic/antigenic variations may also affect the capability of the diagnostic tests and therapeutic agents, most of which are based on WT SARS-CoV-2. Likewise, SARS-CoV-2 literature, particularly on the *in vitro* experiments that is mostly based on WT SARS-CoV-2, may lead to misleading conclusions if being extrapolated to understand the Delta virus biology. Despite several studies on virus isolation (Keyaerts et al., 2005), the precise natures of the kinetics of viral life cycle and nature of CPE produced by WT and Delta variants of SARS-CoV-2 are not well studied. Therefore, we compared the growth characteristics in terms of the kinetics of RNA synthesis, virus production (viral life cycle), plaque formation, and nature of CPE between WT and Delta variants of SARS-CoV-2.

Mutations in envelope proteins of RNA viruses have been shown to be associated with altered viral fitness, eventually affecting the viral plaque morphology (Mandary et al., 2019). In our study, we observed that the Delta variant of SARS-CoV-2 produces a rapid CPE within 24–36 h as compared to 48–72 h by WT. Besides, the Delta variant had bigger plaque size and a shorter life cycle (~ 6 h as compared to the ~8 h in WT). In SARS-CoV-2, D614G or P681R mutations (**Table 1**) were shown to enhance the replication fitness of Delta strain, although no alteration in plaque morphology was observed (Liu Y. et al., 2021; Plante et al., 2021), suggesting that plaque morphology and replication fitness may be determined by independent residues (Liu J. et al., 2021). In addition, the Delta variant rapidly synthesized viral RNA and achieved peak viral titers within 24 h as compared to the 48 h in WT. These observations on faster replication rate of the Delta variant reported in this study support the clinical finding, namely, high transmissibility of Delta variant (Gan et al., 2021; Xu et al., 2021).

Summarily, our neutralization experiments indicated that antibodies elicited by vaccination or infection with WT virus can more effectively neutralize WT SARS-CoV-2 (potential source of the available vaccine) but are significantly less potent against the Delta variant (**Table 2**), the strain that is currently circulating in India. Likewise, W2 sera were more strongly neutralized by Delta as compared to WT SARS-CoV-2 (**Table 2**). These findings on poor cross-neutralization are somewhat in agreement with few other recent findings (Hammerschmidt et al., 2021; Planas et al., 2021).

Exceptionally, two of the sera from vaccinated individuals (**Table 2**; SR134 and SR139), which had no prior history of COVID-19 infection but were diabetic, equally neutralized (antibody titer 128 against each) by both WT and Delta variants. One possibility is that these vaccinated individuals could have been exposed to the Delta virus but did not develop any clinical disease. Although we cannot make any firm conclusions based on mere two samples, immune response against SARS-CoV-2 in diabetic patients could certainly be a matter of further research (Heald et al., 2021a; Heald et al., 2021b; Zheng et al., 2021).

Interestingly, one of the individuals (**Table 2**, SR116A and SR116B) tested positive for COVID-19 during both the first and second waves (also developed clinical disease on each occasion). The antibody titers in this individual were 32 and <8, respectively, against WT and Delta SARS-CoV-2, following 1 month after primary infection, whereas after 18 days following secondary infection, the titers were 64 and 128, respectively. This single but rare sample has again raised concern about the protective efficacy of cross-neutralizing antibodies elicited by vaccination in providing protection against Delta or other newly emerging strains of SARS-CoV-2.

Potential limitations of our work include the low number of infected individuals analyzed and the lack of data on cellular immune responses. Future studies on large number of sera for longer time periods to characterize the role of humoral responses could elaborate on the efficacy of the existing vaccine (prepared from WT SARS-CoV-2) against the circulating variants.

In conclusion, our results demonstrate that Delta variant is poorly neutralized by antibodies elicited by previous infection with SARS-CoV-2 or by vaccination.

## Data Availability Statement

The datasets presented in this study can be found in online repositories. The names of the repository/repositories and accession number(s) can be found below: https://www.ncbi.nlm.nih.gov/genbank/, MW555317.

## Ethics Statement

Samples were collected from patients by the authorized District Medical Officer Hisar, India. A due consent was taken from the patients before collection of the serum samples.

## Author Contributions

Conceptualization: NKu and BT. Formal analysis: NKu and BT. Funding acquisition: NKu and BG. Methodology: NKu, NKh, RK, AV, HN, YC, PM, RT, SV, SKa, and SKh. Writing—first draft: NKu. Writing—review and editing: NKu, NKh, RK, AV, HN, YC, PM, RT, SV, SKa, BT, BG, and YP. All authors contributed to the article and approved the submitted version.

## Funding

This work was supported by Indian Council of Agricultural Research, New Delhi (grant number IXX14586 to NKu and NASF/ABA-8027/2020-21 to NKu and BG) and Science and Engineering Research Board, Department of Science and Technology, Government of India (grant number CVD/2020/000103 to NKu). The funders had no role in the study design, data collection and analysis, decision to publish, or preparation of the manuscript.

## Conflict of Interest

The authors declare that the research was conducted in the absence of any commercial or financial relationships that could be construed as a potential conflict of interest.

## Publisher’s Note

All claims expressed in this article are solely those of the authors and do not necessarily represent those of their affiliated organizations, or those of the publisher, the editors and the reviewers. Any product that may be evaluated in this article, or claim that may be made by its manufacturer, is not guaranteed or endorsed by the publisher.
